# Supplementary material for: Complexome profiling of the Chlamydomonas psb28 mutant reveals TEF5 as an early PSII assembly factor
Source: Plant Cell. 2025 Mar 17;37(6):koaf055. doi: 10.1093/plcell/koaf055 (PMC12164590; doi:10.1093/plcell/koaf055)
Supplement: koaf055_Supplementary_Data [file koaf055_supplementary_data.zip › psb28_interactive.zip!/plots/intensity/qualityPlots/peptides/_Cre10.g423300.t1.1.html]

### Cre10.g423300.t1.1

Trivial name:   
SeqCoverage [median]: 10.60  
Mass: 83.98 kDa  
  
 

| Sequence | MissedCleavage | Mass [Da] | wtIntensity | muIntensity | Score |
| --- | --- | --- | --- | --- | --- |
| AGAAAPAAAK | 0 | 797.44 | 809.53 | 352.32 | 74.25 |
| APGAPVLVTPGSEAR | 0 | 1420.77 | 684.97 | 546.43 | 88.60 |
| LDSGLTSFASGPAVR | 0 | 1476.76 | 2228.63 | 0.00 | 93.35 |
| LLVAVSEVHRDQTSGAELGPDQVVNR | 1 | 2788.45 | 526.00 | 0.00 | 43.89 |
| TGDGEVHDVTPGVDTGFNVR | 0 | 2070.96 | 0.00 | 1717.54 | 52.30 |
|  |  |  | 4249.1 | 2616.3 |  |
